# Comparisons of artificial intelligence automated segmentation techniques to manual segmentation techniques of the maxilla and maxillary sinus for CT or cone-beam CT scans—a systematic review

**DOI:** 10.1093/dmfr/twaf042

**Published:** 2025-06-03

**Authors:** Joon Ha Park, Mustafa Hamimi, Joanne Jung Eun Choi, Carlos Marcelo S Figueredo, Andrew B Cameron

**Affiliations:** School of Medicine and Dentistry, Griffith University, Gold Coast, 4222, Australia; School of Medicine and Dentistry, Griffith University, Gold Coast, 4222, Australia; Sir John Walsh Research Institute, Faculty of Dentistry, University of Otago, Dunedin, 9010, New Zealand; School of Medicine and Dentistry, Griffith University, Gold Coast, 4222, Australia; Department of Dental Medicine, Karolinska Institutet, Stockholm, 17177, Sweden; School of Medicine and Dentistry, Griffith University, Gold Coast, 4222, Australia; Sir John Walsh Research Institute, Faculty of Dentistry, University of Otago, Dunedin, 9010, New Zealand

**Keywords:** artificial intelligence, CBCT, maxillary sinus, segmentation, automatic segmentation, accuracy

## Abstract

**Objectives:**

Accurate segmentation of the maxillary sinus from medical images is essential for diagnostic purposes and surgical planning. Manual segmentation of the maxillary sinus, while the gold standard, is time consuming and requires adequate training. To overcome this problem, artificial intelligence (AI) enabled automatic segmentation software’s developed. The purpose of this review is to systematically analyse the current literature to investigate the accuracy and efficiency of automatic segmentation techniques of the maxillary sinus to manual segmentation.

**Methods:**

A systematic approach to perform a thorough analysis of the existing literature using PRISMA guidelines. Data for this study was obtained from Pubmed, Medline, Embase, and Google Scholar databases. The inclusion and exclusion eligibility criteria were used to shortlist relevant studies. The sample size, anatomical structures segmented, experience of operators, type of manual segmentation software used, type of automatic segmentation software used, statistical comparative method used, and length of time of segmentation were analysed.

**Results:**

This systematic review presents 10 studies that compared the accuracy and efficiency of automatic segmentation of the maxillary sinus to manual segmentation. All the studies included in this study were found to have a low risk of bias. Samples sizes ranged from 3 to 144, a variety of operators were used to manually segment the cone-beam computed tomography (CBCT) and segmentation was made primarily to 3D slicer and Mimics software. The comparison was primarily made to Unet architecture softwares, with the dice-coefficient being the primary means of comparison.

**Conclusions:**

This systematic review showed that automatic segmentation technique was consistently faster than manual segmentation techniques and over 90% accurate when compared to the gold standard of manual segmentation.

## Introduction

Modern dentistry and craniofacial surgery have embraced the digital workflow utilising various diagnostic tools including: X-rays, multi-slice computed tomography (MSCT) scans, intra-oral scanners and cone-beam computed tomography (CBCT) scans.^1^ In addition, the use of manual and semi-manual segmentation software allows the operator to transfer Digital Imaging and Communications in Medicine (DICOM) data from CBCTs and MSCTs to create a “virtual patient”.^2^ This allows the manipulation and interpretation of parts of patient’s craniofacial structures digitally.^2^^,^^3^ This has led to an increase in accuracy in surgical planning, designing surgical guides and defining surgical margins.^4–6^ However, using manual segmentation on craniofacial structures involves delineation of bone contours multiple times on multiple CT slices by the operator.^6^

The operator needs to be adequately trained to perform the segmentation otherwise it can lead to operator dependent bias.^7^^,^^8^ Moreover, the operator also needs to have the anatomical knowledge of the craniofacial area since there are multiple bones that are in contact and identifying sutures between different bones are sometimes difficult to interpret.^9^ Materials with high densities such as metal crowns, amalgam restorations and orthodontic brackets can also cause “artefacts” on MSCT/CBCT images which could disrupt the interpretation and segmentation of surrounding structures and cause difficulty in segmentation.^9^^,^^10^ Manual segmentation is, therefore, time consuming, tedious, and a relatively inefficient process.^11^

The introduction of artificial intelligence (AI) enabled automatic segmentation into the workflow in the field of dentistry and craniofacial surgery is a rapidly developing area in dentistry.^9^^,^^12^^,^^13^ AI has a potential to aid clinician’s diagnosis with more efficient and accurate analysis of medical images, such as CBCT’s, which allows streamlining of the clinical diagnosis.^3^ AI segmentation has been shown to provide better outcome for patients and better workflow for practitioners. An example can be seen in oncological radio-neurosurgery where automated detection and segmentation of brain tumours using artificial intelligence has shown to increase efficiency and accuracy in tumour diagnosis, saving time in tumour contouring for radiation planning and reduce inter-practitioner variability.^14^ Moreover, AI segmentation technology was able to monitor treatment responses and detect tumour recurrence such as detecting brain metastases and gliomas. Other benefits may include the elimination of artefacts generated during image capture and eliminate variability in operator interpretation error due to the consistent and large number of data sources it can draw from in an unbiased context.^15^^,^^16^ However, AI automatic segmentation is still a developing field with advances occurring at a rapid pace. There are several applications available in the market which has not been validated for reliability with independent investigation.^17^

One of the key factors in determining if manual segmentation or AI segmentation is a clinically acceptable diagnostic tool is how accurately different anatomical structures with different radio densities can be segmented. Inaccurate segmentation can lead to poor interpretation of clinical findings or misguided clinical procedures.^18^^,^^19^ A variety of methods have been utilised to compare the quality of segmented data from CBCT or MSCT.^18^^,^^20^^,^^21^ These range from comparing the volume of a pathology or anatomical feature,^5^^,^^15^ surface deviation analysis with metrology software,^15^^,^^22^ intersection-over-union scores (used in object category segmentation) for the mandible and tooth structures,^23^ and the use of dice score coefficient to compare segmented regions of anatomy.^24^ The variety of methods used to assess accuracy of segmented anatomical structures can make comparisons of studies problematic. Furthermore, the use of only one of these techniques may may limit the reliability of the results of individual studies.

AI segmentation has been shown to provide better outcome for patients and better workflow for practitioners. For example, in oncological radio-neurosurgery, automated detection and segmentation of brain tumours using artificial intelligence has shown to increase efficiency and accuracy in tumour diagnosis, saving time in tumour contouring for radiation planning and reduce inter-practitioner variability. Moreover, AI segmentation technology was able to monitor treatment responses and detect tumour recurrence such as detecting brain metastases and gliomas.^14^

The evidence that AI enabled automatic segmented applications can identify the varied range of anatomical structures in the head and neck is an emerging area of investigation. In addition to this, there are number of anatomical structures that can be segmented out from the craniofacial region, this includes, but not limited to frontal bone, nasal bone, orbits, maxilla, mandible, zygoma, upper and lower dentitions as well as a range of known pathologies.^2^^,^^6^^,^^9^^,^^25–29^ More specifically in the craniofacial region, there has been more focus in areas such as the dentition, the mandible and the inferior alveolar nerve due to their close proximity to each other and clinical relevance in many dental specialities.^17^^,^^23^ Systemic review focusing on these anatomical structures have already been published and past studies have been reviewed and summarised indicating that there is an emerging volume of research on this topic.^2^^,^^20^^,^^30^ However, despite research in automatic segmentation of maxillary sinus being evident, no systematic literature review on this anatomical region exists. This systematic literature review looks to answer the question: Are AI segmentation techniques comparable to manual segmentation techniques from the perspectives of accuracy and effectiveness for the maxillary sinus? The aim of this systematic review was to investigate how past studies compare AI enabled automatic segmentation of the maxillary sinus to manual segmentation techniques.

## Methods

### Search strategy and search terms

The proposed systemic review has been conducted in accordance to Preferred Reporting Items for Systemic Reviews and Meta-analyses (PRISMA) guidelines.^31^ In order to answer our research question pertaining to articles related to automatic maxillary sinus segmentation using artificial intelligence (AI), a search strategy using medical subject headings (MeSH) terms and free words pooled through a Boolean operators (‘AND’, ‘OR’) on the following databases: Pubmed, Medline, Embase. A customised search strategy using MeSH keywords “cone beam computed tomography” AND “maxillary sinus” OR “antrum, maxillary” AND “segmentation” was implemented in each database, shown in Table 1. Additionally, Scopus and Google Scholar were also used to expand and validate our search.

**Table 1.** List of search terms for each database used.

PubMed	((((segmentation) AND (cone beam computed tomography[MeSH Terms])) AND (maxillary sinus[MeSH Terms]) OR (antrum, maxillary[MeSH Terms]) AND (2007:2024[pdat]) AND (2022:2024[pdat])))
MedLine	((Tomography, X-ray computed OR Cone-Beam Computed Tomography) AND (Maxilla OR Maxillary Sinus) AND (Deep Learning OR Neural Networks, Computer OR Artificial intelligence or Image processing, computer-assisted) AND (Segmentation))
Embase	(((CT scanner OR cone beam computed tomography) AND (maxillary sinus OR maxilla)) AND ((deep learning algorithm OR artificial neural network OR artificial intelligence software)) AND (segmentation))
Scopus	automatic AND segmentation AND maxilla
Google Scholar	automatic segmentation of maxillary sinus

Search term “automatic AND segmentation AND maxilla” was used in Scopus with time range of 2020-2024. The search term: “automatic segmentation of maxillary sinus” was used in Google Scholar with time range of 2020-2024. Above search was carried out until September 2024.

### Selection criteria

The inclusion criteria adopted for articles selection were: English language only, full length peer-review, Maxillary sinus without any artefacts, abnormalities, or pathologies while able to delineate all the margins, bilateral max sinus available on computed tomography scan and segmentation tool based on fully automatic algorithm. The exclusion criteria included: foreign languages other than English, segmentation that does not use deep learning algorithm, use of imaging modalities other than CT or CBCT, images that has maxillary sinus with abnormalities such as lesions or artefacts that could interfere with delineation of the margins of the maxillary sinus. Two authors (JP and MH) have cross checked each other’s article and if any problem was found, the third author JC was contacted for review. Further details are outlined in Table 2. Inter-rater reliability between the three authors was evaluated using PRISM V10 using Cohen’s Kappa Coefficient which met satisfactory threshold before a risk of bias assessment was conducted.

**Table 2.** Inclusion and exclusion criteria.

Inclusion criteria	Exclusion criteria
English language. Maxillary sinus without any artefacts, abnormalities, lesions and able to delineate all the margins of maxillary sinus, bilateral max sinus available on CT. Segmentation tool based on fully automated algorithm.	Foreign language other than English. Semi-automatic or manual segmentation, automatic segmentation without using deep learning algorithm. Imaging modalities other than CT or CBCT (e.g. OPG). Maxillary sinus with abnormalities such as lesions or maxillary sinus not fully delineated or disrupted by artefacts. No conference reports. No opinion articles.

### Risk of bias assessment

The risk of bias was performed by two independent reviewers (JC and MH) using Modified Consolidated Standards of Reporting Trials (CONSORT),^32^ which is composed of six domains: abstract, introduction, methods, results, discussion and other information. When required, a third review author has been involved in meditating any disagreement between the two independent reviewers regarding the risk of bias assessment in certain articles, this happened on two occasion and was mediated by JC. See Table 3 for further details.

**Table 3.** A modified GRADE/CONSORT-2 risk of bias table.

	Abstract	Introduction Background	Introduction Objectives	Methods Intervention	Patient Selection (data set)^a^	Index test^a^	Reference standard^a^	Methods Outcomes	Methods Sample Size	Methods: Statistical methods	Results: Outcomes and estimation	Discussion: Limitation	Other information: Funding	Other information: Protocol	Overall Risk of Bias
Xu et al (2020)	Yes	Yes	Yes	Yes	High risk	High risk	Low risk	Yes	No	No	Yes	Yes	Yes	No	Low
Park et al (2021)	Yes	Yes	Yes	Yes	Low risk	Low risk	Low risk	Yes	Yes	No	Yes	Yes	Yes	No	Low
Yang et al (2021)	Yes	Yes	yes	Yes	High risk	Unclear	Unclear	Yes	Yes	No	Yes	Yes	Yes	Yes	Low
Morgan et al (2022)	Yes	Yes	Yes	Yes	High risk	Low risk	Low risk	Yes	Yes	No	Yes	Yes	Yes	Yes	Low
Preda et al (2022)	Yes	Yes	Yes	Yes	High risk	Low risk	Low risk	Yes	Yes	Yes	Yes	Yes	Yes	Yes	Low
Nogueira‐Reis et al (2022)	Yes	Yes	Yes	Yes	High risk	Unclear	Low risk	Yes	Yes	Yes	Yes	Yes	Yes	Yes	Low
Choi et al (2022)	Yes	Yes	Yes	Yes	High risk	Low risk	Low risk	Yes	Yes	No	Yes	Yes	Yes	Yes	Low
Morita et al (2022)	Yes	Yes	Yes	Yes	High risk	Low risk	Low risk	Yes	Yes	No	Yes	Yes	Yes	no	Low
Yoo (2023)	Yes	Yes	Yes	Yes	High risk	Low risk	Low risk	Yes	Yes	Yes	Yes	Yes	Yes	Yes	Low
Bayrakdar (2024)	Yes	Yes	Yes	Yes	High Risk	Low risk	Low risk	Yes	Yes	Yes	Yes	Yes	Yes	Yes	Low

a Denotes parameters assessed from the CONSORT-2 risk of bias.

### Data collection and analysis

An excel spreadsheet (Microsoft Corporation, Redmond, USA) was made and one reviewer (JP) in the presence of a second reviewer (MH) extracted the data, while two reviewers (JP and MH) examined it. All potential studies were imported into a reference manager (Endnote V22, Clarivate analytics, Philadelphia, USA). Duplicates were removed. The papers were first screened via title resulting in 142 paper, then screened via abstract resulting in 22 papers. A full text review was then conducted on the remaining papers resulting. A total of three papers were included that JP and MH were not on agreeance on. These discrepancies were resolved with discussion between three reviewers (JP, MH and JC). The data collection included in the study included study information (authors, year, journal and title), sample size, segmented anatomical structure, operators, segmentation software (manual and automatic), algorithm used, comparative method, time to segment (manual and automatic) and key findings.

## Results

### Search and selection process

**Figure 1.**
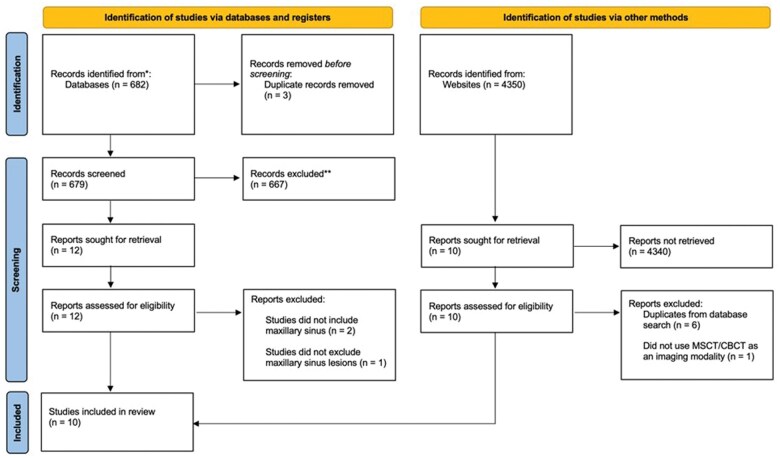
PRISMA2020 flow diagram of the search strategy and results.

The search strategy identified 5032 articles through the different databases: 635 from Pubmed, 13 from Medline, 9 from Embase, 25 from Scopus and 4350 from Google Scholar. Nine duplicate articles were removed. Five thousand and thirty two articles were screened for title and abstract. The process led to 15 studies eligible full text evaluation. There was a disagreement between JP and MH which was resolved by JC and both with both papers being included. This resulted in an inter-assessor reliability value (Cohen’s kappa) of 0.98. A further four articles were excluded for different reasons: One study was excluded due to use of Orthopantomagram (OPG) as an imaging modality instead of MSCT/CBCT.^33^ Another study did not exclude data that contains maxillary sinus lesion.^34^ Two studies were excluded since the maxillary sinus was not part of the segmentation.^35^^,^^36^ Ten studies were included in this systemic review for qualitative synthesis.^4^^,^^13^^,^^27–29^^,^^37–41^ The PRISMA 2020 flow diagram of the search strategy is shown in Figure 1.

### Study characteristics

The results from Table 1 show considerable variation in the parameters used in studies. The smallest sample size was 3 patients, while the highest number of patient CBCT scans was 144.^4^^,^^13^ The limited number of sample sizes in the study containing 3 samples may negate its validity.^4^ The remaining studies do present data with sample sizes starting at 45. This would indicate that this study with minimal data may be viewed in terms of only developing a technique and not informing researchers and clinicians on the accuracy of the technologies implemented. One study used publicly available data sets.^41^ Only three of the studies included gender distribution of sample participants.^28^^,^^39^^,^^40^ The most popular manual segmentation software used were 3D slicer and Mimics software.^4^^,^^13^^,^^28^^,^^39–41^ U-net architecture was most popular AI algorithm architecturef used.^27^^,^^38–41^ The anatomical structures segmented across the studies includes the maxillary sinus, mandible, maxilla, skull, upper skull, zygomaticomaxillary complex, maxillary teeth, mandibular teeth, nasal, frontal bone, nasal bone and zygoma. Only four of the studies mentioned training data sets in their study.^27^^,^^37–39^ The operators used in these studies varied in profession. They include radiologists, oral and maxillofacial surgeons, biomedical engineers, radiographers, radiation oncologists, dento-maxillary radiologists, oral surgeons and plastic surgeons. Given the wide range of specialties of the assessors this may lead to different degrees of segmentation based on the operators past experience or level of radiology training. The different operators that performed the manual segmentation are highlighted in Table 4. It should also be notes that of the 10 studies included in the systematic review only 7 included direct comparisons between manual and AI segmentation methods.

**Table 4.** Summary of selected articles and their characteristics.

Author (Year)	Sample size	Anatomical structure segmented	Operators	Manual segmentation Software	Automatic segmentation
Xu (2020)	61 CTs	Max sinus - 61 (35 training, 26 for validating and testing)	Two experienced doctors One radiologist	Manual segmentation using ITK-SNAP 3.8	VGG network
Park et al. (2021)	4 data sets. Centre A (15 MDCT), Centre B (15 MDCT), PDDCA (28 MDCT), and TCIA (15 CBCT). PDDCA (Public Domain Database for Computational Anatomy) and TCIA (The Cancer Imaging Archive) are publicly available datasets	Mandible and maxilla. Training data sets- 146. validation data sets-10 Test data sets- 15.	Centre A Oral and maxillofacial surgeon Centre B Biomedical engineers supervised by a clinical expert TCIA and PDDCA Radiographer and radiation oncologist	N/A	Pre-processed scan input in 2.5D. HPMR compared to Unet and Res-U-net. Using Pytorch framework in Python
Yang et al (2021)	Pilot study 3 patients- no mention of number of CTs	Skull CT scan. No mention of training, validation or test data set number. Maxilla, mandible, upper teeth, lower teeth, neck segmented.	N/A	ProPlan CMF 2.0 (Materialise)	Mimics viewer
Morgan (2020)	132 CBCTs - 75 Females 57 males	Max sinus - 132 (83 training, 19 validation, 30 testing)	One Dentomaxillary radiologist	Mimics software	Unet/Virtual patient creator (Relu)
Preada (2022)	144 CBCTs	Upper Skull - 30 ZMC - 80	One orthodontist One Maxillofacial radiologist One Oral surgeon	Mimics innovation suite	Virtual patient creator (Relu)
Nogueira‐Reis et al (2022)	40 CBCTs	ZMC - 40 Maxillary sinus - 80 Max teeth - 560	Two dentomaxillofacial radiologist	No manual segmentation to compare with automatic segmentation	Virtual patient creator
Choi et al (2022)	45 CBCTs (26 female, 12 male)	Maxillary sinuses - 45 (pair)	One Oral radiologist	3D slicer	U net architecture
Morital et al. (2022)	50 Face CTs	Nasal maxilla mandible R) zygoma L) zygoma Frontal Max teeth Mand teeth	One Plastic surgeon	3D slicer software	U-net architecture
Yoo (2023)	67 patients. 46 females and 21 males. No number of CBCT scans mentioned	Maxillary sinus and maxillary sinus injuries	One oral and maxillofacial radiologist	3D slicer for windows	compared 2D, 2.5D and 3D network using standardized memory capacity
Bayrakdar (2024)	101 CBCTs	Max sinus - 101 (80 train model, 11 validate, 10 to test)	Two maxillofacial radiologist	Craniocatch annotation tool software (Turkey)	− U net architecture algorithm

**Table 5.** Summary of algorithm, methods, time of segmentation and key findings.

Author (Year)	Manual segmentation application and Algorithm used	Comparative method	Time (automatic)	Time (manual)	Outcome (comparison of AI and manual segmentation accuracy—similar, or inferior)	Key Findings
Xu 2020	V-net	Dice coefficient (Dice), intersection over union (Iou), and precision	<1 minute	>15 minutes	similar	DSC - 94.4% (Dice similarity Coefficient)
Park et al. 2021	HPMR Unet, Res-U-Net, mU-Net,	Dice coefficient- Hausdorff distance, average surface distance.	no mention	No mention	N/A	HPMR-U-Net has better segmentation preformation than other models
Yang et al (2021)	AI- Mimics (materialise). Manual segmentation- Pro PLan CMF 2.0	Dice coefficient	time not calculated	Time not calculated	similar	Dice coefficient- 92.4% for maxilla. 94.9% for mandible
Morgan (2020)	2x 3D U-net	Accuracy (trueness or precision?): DSC, Intersection over Union (IoU), Hausdorff distance (HD), Root mean square distance	Automatic - 24.4 secs	Semi-automatic - 60.8 mins	similar	DSC - 98.4%
Preada (2022)	Manual segmentation—Mimics AI—CNN based model - 3D U-net + Virtual patient creator (relu.eu)	Accuracy: DSC	Automatic segmentation: 39.07 sec	Manual segmentation: 132.7 min	similar	204-fold time reduction for the segmentation task compared to the manual approach and the integration of the model into an online platform CNN model has high accuracy
Nogueira‐Reis et al (2022)	Virtual patient creator (relu.eu) -Convolutional neural network	Sample size—G*power 3.1 Accuracy: DSC	Automated segmentation - 1.7 min (average) Refined segmentation - 3.4 mins (average)		N/A	The three integrated CNN models proved to be fast and accurate for simultaneous segmentation of maxillary anatomical structures with different densities.
Choi et al (2022)	U-net	Accuracy: DSC	Automatic segmentation: 46.2 secs	Manual segmentation: 48.7 min	Similar	Mucosal inflammation causes difficulty to segment maxillary sinuses due to mucosal thickening post processing required to remove the false positive
Morital et al. (2022)	Manual segmentation - 3D slicer AI - (2D) U-net architecture	Accurancy 1. Dice similarity co-efficient (DSC) 2. average symmetric surface distance (ASSD)	no mention of time	No mention of time	Similar (mandible and zygomatic bones) Inferior (tooth structures)	Automatic segmentation using U-Net architecture High accuracy—for mandible and zygomatic bones Lower inferiority—teeth
Yoo (2023)	2D used U-net, U-net++ with ResNet101 and DenseNet169. 2.5D used same at 2D network, images analyzed in axial, sagitatal and coronally planes seperately. 3D used 3D u-net, 3D V-t	Jaccard coeffient, dice similarity coeficient, precision	FPS (Frames per second) 2D network Unet- 134 Unet++- 88 2.5D network Unet- 41 Unet++- 24 3D network Unet- 142 Unet++- 447	not mentioned	N/A	3D networks not superior than 2.5D under constant GPU memory.- more false negatives. 2.5D is the most accurate for segmentation
Bayrakdar (2024)	nnU-net	Accuracy: DSC, Hausdoff distance, intersection over union	not mentioned	N/A	Similar	Automatic segmentation is highly accurate DSC - 95%

Due to the diversity in software applications, study designs and outcomes presented and meta-analysis was deemed to be unsuitable for the current systematic review.

### Risk of bias assessment

Ten articles were reviewed for risk of bias in line with the GRADE protocols. Additional analysis was performed with components of the CONSORT-2 protocols as it was deemed that the included studies needed additional analysis due to the nature of the experiments. The result of assessment is presented in Table 3. Six out of ten articles did not show statistical methods.^4^^,^^37–40^ Studies that did demonstrate statistical methods were evaluated for showing descriptive statistics, post-hoc analysis and normality of the data. The objectives of the studies included helped to determine if they were of clinical relevance with a ‘Yes’ in this column indicating they were. The parameters defining patient selection, index test and reference standard from the CONSORT-2 tool were used to enrich the evaluation of the methods of the included studies. These were evaluated as ‘high-risk’, ‘low-risk’ or ‘unclear’. Most of the studies indicated a high risk of bias on data set selection as the majority of data set was from a single institution and had a relatively small sample size that could result in overfitting.^13^^,^^28^^,^^29^^,^^37^^,^^40^^,^^41^ A low risk of bias on index testing was observed as they had clearly outlined model details such as AI architecture, training process and validation strategy. Two studies had used commercially available AI segmentation software for automatic segmentation it was not possible to make a decision on the risk of bias on index testing.^4^^,^^29^ There was no mention of the use of validation set for one study which could have increase the risk of overfitting.^37^ In addition, three of the studies did not report the protocol used.^32–34^ However, overall, all studies showed a low risk of bias.

#### Sub-themes in selected publications

The most popular manual segmentation software used were 3D slicer and Mimics software.^4^^,^^13^^,^^28^^,^^39–41^ Other manual segmentation softwares used included ITK-SNAP 3.8, ProPlan CMF 2.0, and Craniocatch annotation software, these can be seen in Table 5.^4^^,^^27^^,^^37^ Two of the studies did not report the manual segmentation softwares used.^29^^,^^38^ U-net architecture was most popular automatic segmentation software algorithm used.^27^^,^^38–41^ Other automatic segmentation software used included VGG network, and Relu.^37^^,^^39^

The dice-coefficient (DSC) was the most used comparative method to compare segmentation.^4^^,^^27^^,^^37^^,^^39^ Other comparative methods used in combination with DSC or by themselves include the intersection over union, Hausdorr distance, average symmetric surface distance, and the Jaccard coefficient.^28^^,^^37–39^^,^^41^

The fastest time to automatically segment anatomical structures was 39.07 seconds, while the slowest time was 60.8 minutes.^13^^,^^39^ The quickest manual segmentation time was 15 minutes, while the slowest manual segmentation time was 132 minutes.^13^^,^^37^ Several studies did not report automatic or manual segmentation time.^4^^,^^27–29^^,^^38^^,^^41^

## Discussion

Comparisons between automatic segmentation versus semi-automatic/manual segmentation indicate that automatic segmentation models showed remarkable performance over semi-automatic/manual segmentation.^13^^,^^37^^,^^39^^,^^40^ Morgan et al demonstrated that automatic segmentation was 149 times faster than the semi-automatic approach.^39^ This is further supported with Preda et al reporting a 204 times decrease in the time required to segment compared to manual segmentation.^13^ In practical diagnostic terms, in a clinical, setting automatic segmentation has been shown to be a practical option for clinician. The actual time reductions demonstrated by V-net shows that automatic segmentation using V-net took less than one minute compared to a manual segmentation which took more than fifteen minutes.^37^ These studies have shown that use of automatic segmentation in the daily clinical settings can be highly effective in terms of time efficiency whereas performing manual segmentation may not be time-efficient or practical due to significant increase in time consumption in segmentation, requiring highly experienced operator and significant time usage due to correcting operator induced errors.^42^

The studies included in this systemic review has implemented various comparative tools to compare between automatic segmentation and semi-automatic/manual segmentation.^4^^,^^13^^,^^27–29^^,^^37–41^ However, one common tool used in all studies to measure the accuracy of the AI segmented structure was the dice similarity co-efficient (DSC). DSC measures the space overlap between the ground truth and AI segmented structure.^12^ All ten studies included in this systemic review has shown that AI segmentation of various craniofacial structure has >90% DSC. Additionally, one study has demonstrated a clinical evaluation of their AI segmented structure. The study has shown that almost 70% of testing set was classified as a perfect segmentation, with no refinement needed. Moreover, cases that required refinement was due to mucosal thickening of the maxillary sinus. No deficient or negative prediction was present.^39^

The value of accuracy of automatic segmentation provided by these studies either by DSC or clinical evaluation using their own classification criteria has definite clinical relevance. However, the degree of accuracy required depends on clinical situation. For example, patient undergoing removal of cancerous tumour may require higher value of accuracy to completely remove the margins of the tumour.^43^ For this reason, the clinical procedure to be performed should be taken into account when relying on automatic segmentation for clinical diagnostic purposes.

CBCT images of the maxillofacial regions allow the inspection of the entire volume of the maxillary sinus. However, it was found that oral radiologist who interpret the CBCT images often neglect the abnormalities within the maxillary sinus region.^44^ We hope that AI segmentation of maxilla in the future will provide “safe-netting” of these findings and aid clinicians to detect any subtle abnormalities of the maxillary sinus. Another potential clinical use case is to observe volumetric bone changes after sinus floor augmentation.^45^ Mineralised cortical bone can be placed prior to implant placement in the maxilla and by using AI maxillary sinus segmentation, we can observe the volume changes within the floor of the maxilla and therefore help with the prognostication of implant placement.

The articles included in this systemic review have used a variety of operators for its manual segmentation. It ranged from dental professionals of different dental specialitie to a medically trained specialist.^4^^,^^13^^,^^27–29^^,^^37–41^ These included plastic surgeons^41^ and radiologists^37^^,^^40^ and a biomedical engineer who was supervised by a clinician.^38^ The accuracy of the manual segmentation highly depends on the operator’s experience in manual segmentation.^37^ However their experience in manual segmentation varied,^27^^,^^39^^,^^40^ or the study did not mention how experienced the operator was.^4^^,^^13^^,^^28^^,^^38^^,^^41^ Different type of operators with various experiences in manual segmentation could results in variation to the “ground truth” where automatic segmented structure is compared to. The accuracy of the automatic segmentation depends on this manual segmentation as the ground truth, as the statistically validation tool such as DSC which measures the space overlap between the ground truth and AI segmented maxillary sinus.^12^ If this is not accurate the results of a study may be invalid.

Most of the studies, included in this systematic review, have used U-net architecture convolutional network.^27^^,^^38–41^ Some studies opted to created their own training model and use their own training set,^27^^,^^28^^,^^37^^,^^38^^,^^40^^,^^41^ validation set and test data set where as other studies has used a software from third parties such as the virtual patient creator (Relu, Leuven, Belgium). The question remains whether these trained models are medically compliant in different countries to be used in clinical settings. Moreover, each study has different set of training set, validations set and testing data set. Whether this has any impact on the accuracy of the segmentation and clinically relevance remains to be investigated. Further research is needed to compare accuracy between automatic segmentations from self-trained model vs readily available software such as the virtual patient creator.

Currently the virtual patient creator has certification in ISO13485 with CE and 510(K) from FDA.^46^^,^^47^ The mimics software (Materialise NV) also has ISO 13485 with CE and 510(K) pre-market clearance from FDA.^47^^,^^48^ However, the self-trained AI model based on convolutional neural network (CNN) and U-net architecture does not have approvals for medical us in most jurisdictions.^47^ Further research and validation from the regulatory body will be required to use these models in the clinical settings. There has been reported issues with segmentation of the maxillary sinus using CNN model. Some studies have reported that CNN model can extract the normal clear sinus and separate the bony boarders. However, it could not delineate the soft tissues.^39^ This could be deemed as a significant problem when there is a pathology within the maxillary sinus cavity. Another study has reported that the accuracy of maxillary sinus segmentation with mucosal inflammation is unsatisfactory when using V-net.^37^ Moreover, false positive pixels were observed while using U-net model on segmentation of maxillary sinus. The author has suggested that further processing is required to detect the edges of maxillary sinus.^40^

With the advent of artificial intelligence (AI), there has been number of instances where the general public has been using this technology to self-diagnose a medical condition.^49^ There is a potential risk that these AI segmentation software which is not validated by a various regulatory body can be used by the general public wrongly which can create risks when diagnosis has not been proven to be accurate.^50^

The accuracy of automatic segmentation needs to be tested in wide range of clinical settings. The future research may include craniofacial structures with pathologies and further testing with CNN models. Different methodological approaches to assessing the segmentation should also be performed other than relying on the DSC coefficient. DSC coefficients could be compared to intersection-over-union scores to validate the results.^23^ In addition, a variety of metrology techniques could be applied in this research field. These include the use of alignment of the reference data set (manual segmentation) to the measured (AI segmentation) then a 3D comparison comprising of positive and negative deviations in different regions, a technique used extensively to test the accuracy of restorative dental materials.^51^^,^^52^ Furthermore, a validation of different manual segmentation techniques and software applications would aid in validating what is perceived to be the actual anatomical feature for these types of studies. This is required to achieve and validate the accuracy that is satisfactory for clinical use of these software when used for diagnosing and surgical planning purposes. Comparative study between different third-party software should be done to compare their accuracy, consistency, time efficiency and cost-effectiveness in daily clinical settings.

The limitations of this systematic review include the lack of generalisability of the models. Most of the studies have used dataset from a single institution.^13^^,^^27^^,^^28^^,^^37^^,^^39^^,^^41^ Moreover, the use of single CBCT/MSCT device led to lack of data heterogenicity.^13^^,^^27^^,^^28^^,^^37^^,^^39^^,^^41^ Future studies should investigate using different CBCT/MSCT devices with different parameters to solve the above issues. One study has mentioned that they weren’t able to remove the false positive pixels when its connected to the maxillary sinus region during post processing.^40^ This could be a major flaw to the model when applying this technique for planning surgery or treatment. There needs to be a process required to detect the edge of the maxillary sinus. Some models were able to delineate between bony borders in case of sinus thickening in maxillary sinus. However, it couldn’t delineate a soft tissue.^39^ Again, this has implications in the presence of maxillary sinus pathologies. There needs to be a model to delineate soft tissue pathologies for the model to be used for surgical applications.

To increase the generalisability of the model, the future model should set the training data set and testing data set to 50-50%. Equal distribution of training and testing data set will allow better accuracy on evaluating its performance. If one class is overrepresented, this could result in overfitting of that class. Moreover, balanced training and validation data could allow increased reliability on real-world data.^53^ Only one study had no clear explanation of reference standards on ground truth.^4^ It did not mention who the operators were on manual segmentation and their experience in the field nor explained their skill set on maxillofacial segmentation.

## Conclusion

Our study showed that automatic segmentation technique was consistently faster than manual segmentation techniques and over 90% accurate when compared to the gold standard of manual segmentation. Current shortcomings within the field include potential for operator error, software validation and FDA/CE approval, and accuracy and clinical translatability of automatic segmentation software’s for diagnostic purposes.
